# Hypokalemia-Induced Rhabdomyolysis From Budesonide Therapy in Crohn's Disease

**DOI:** 10.14309/crj.0000000000000201

**Published:** 2019-08-29

**Authors:** Ramy Mansour, Pujyitha Mandiga, Derek Thigpin

**Affiliations:** 1 Department of Medical Education, Gastroenterology, Ascension Genesys, Grand Blanc, MI; 2 Department of Medical Education, Internal Medicine, Ascension Genesys, Grand Blanc, MI

## Abstract

We present a 71-year-old white man with active ileocolonic Crohn's disease, recently started on budesonide therapy, who presented with extreme weakness and muscle aches. He was diagnosed with hypokalemia-induced rhabdomyolysis, 3 weeks after starting budesonide therapy. His symptoms and laboratory values improved with budesonide discontinuation and appropriate fluid and electrolyte replacement. This is only the second case of hypokalemia-induced rhabdomyolysis secondary to budesonide.

## INTRODUCTION

Budesonide has long been accepted as induction therapy for mild to moderate Crohn's disease (CD).^1^ Although budesonide is considered highly efficacious because of its anti-inflammatory properties, it can be offsetting because of its serious side effects.^2^ We present only the second reported case of hypokalemia-induced rhabdomyolysis secondary to budesonide.

## CASE REPORT

A 71-year-old white man presented with hypertension and ileocolonic stricturing CD since 1974. He had undergone 3 surgeries including ileocecectomy and 2 small bowel resections in 1975, 1986, and 2007. Current medications include 6-mercaptopurine, amlodipine, metoprolol, vitamin D, calcium, and a multivitamin. He was previously treated with sulfasalazine and oral mesalamine. He sparingly required oral steroids throughout his life. In October 2018, he presented for a 5-month history of worsening watery diarrhea and unintentional weight loss. Stool studies, including *Clostridium difficile*, were negative. Colonoscopy to the neoterminal ileum revealed a Rutgeerts score of i3. He was started on oral budesonide as a bridge to biologic therapy.

After starting 9 mg of budesonide, the patient immediately began to experience weakness and muscle aches. Three weeks later, these symptoms progressed and he was unable to walk or lift his arms above his head. He presented to the hospital, and laboratory tests revealed magnesium 0.8 mg/dL, potassium 1.7 mmol/L, creatinine 1.52 mg/dL, creatine phosphokinase 15,913 IU/L, calcium 7.1 mg/dL, albumin 3.7 g/dL, aspartate aminotransferase 253 U/L, alanine aminotransferase 125 U/L, and lactate dehydrogenase 734 IU/L. Urinalysis showed 3+ blood and 0–3 red blood cells. Laboratory values 2 months before were unremarkable, despite having chronic diarrhea for several months (Table 1). He was diagnosed with rhabdomyolysis. Intravenous fluids and electrolyte replacement were initiated. The patient felt that the onset of symptoms correlated with the initiation of budesonide, so the medication was held. Over the course of the week, his symptoms improved drastically and ultimately resolved, correlating with creatine phosphokinase and potassium improvement. He followed up 3 weeks later, asymptomatic and with normal laboratory values (Table 1).

**Table 1.**
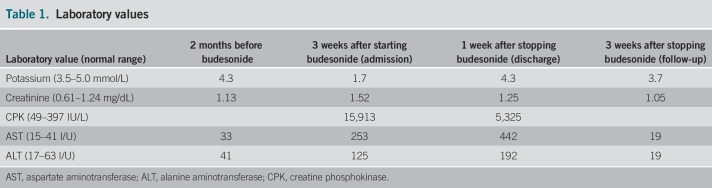
Laboratory values

## DISCUSSION

Hypokalemia-induced rhabdomyolysis due to laxative abuse, chronic diarrhea, renal tubular acidosis, and diuretics use is well reported.^3,4^ Hypokalemia is a known side effect of budesonide; however, there has only been 1 case report of hypokalemia-induced rhabdomyolysis from budesonide therapy.^1,5,6^ Rhabdomyolysis is a syndrome which is characterized by muscle necrosis with creatine phosphokinase 4 times more than the upper limit of normal.^4^

Hypokalemia is usually asymptomatic unless the potassium is less than 2.5 mEq/L, at which point it can cause severe muscle necrosis leading to rhabdomyolysis (7). Long-term diarrhea due to an acute CD flare can itself lead to several electrolyte deficiencies such as hypokalemia and metabolic acidosis because of loss of potassium from the colon and bicarbonate ions from gastrointestinal tract. Other common causes of hypokalemia include short bowel syndrome, Conn syndrome, renal tubular acidosis, Gitelman syndrome, Bartter syndrome, pheochromocytoma, alcoholism, and multiple medications.^8^

Budesonide is a corticosteroid, which structurally is closer to 16-alpha hydroxyprednisolone, and has a strong affinity to corticosteroid receptors with hepatic metabolism, causing minimal side effects.^9^ Budesonide is available in controlled release enteric formulation, which works mainly on the distal ileum and ascending colon. According to the studies, 52%–79% of the drug is absorbed, but the systemic bioavailability is only 9.3% on average.^10^ The use of budesonide has become more popular over other systemic glucocorticoids such as prednisone because of the lower incidence of systemic side effects. Studies showed that side effects seen in patients were 16%–33% with budesonide when compared with 55% with prednisone use.^1,2^ Although there are many reported side effects of budesonide, hypokalemia is rarely seen in these patients.^1,5^

Corticosteroids regulate the fluid and electrolyte homeostasis in the body. They stimulate sodium absorption in the small intestine and the colon while promoting potassium secretion from the kidney, colon, and salivary glands because of their mineralocorticoid action. Chronic administration of corticosteroids in vivo produced similar results of sodium absorption and potassium secretion because of the Na+/K+-ATPase activity, leading to mild hypernatremia and severe hypokalemia.^11^

During a muscle contraction, potassium is released from the intracellular to the extracellular space, mediating the vasodilation and more blood flow to the muscle. When a patient has hypokalemia, there is a decrease in blood flow to the contracting muscles. This leads to constriction of arterioles, causing ischemia of the muscles and myocytes destruction. This causes decreased glycolytic enzyme function and increased accumulation of free fatty acids into the myocytes, leading to a dysfunction of the Na/K-ATPase, Ca2+-ATPase pump. This increases the cellular membrane permeability and intracellular calcium concentration, which stimulates the proteases and phospholipases leading to the destruction of the myofibril, cytoskeletal, and proteins. The breakdown of the myofibril causes muscle necrosis and an increase in intracellular CPK and myoglobin, which is released into the blood circulation.^12^

In this patient, several factors were taken into account for the cause of hypokalemia-induced rhabdomyolysis. One possibility was Bartter syndrome, which is a known cause of extreme hypokalemia leading to rhabdomyolysis.^13^ However, the patient rapidly responded to intervenous fluid and discontinuation of the budeosnide, making Bartter syndrome less likely. The patient had chronic diarrhea for 5 months, which could have led to hypokalemia contributing to his generalized fatigue, although his potassium was normal while he had diarrhea just 2 months before starting therapy (Table 1). It is possible that the chronic diarrhea made him susceptible to another cause of hypokalemia. Symptom onset correlated well with budesonide initiation, and the symptoms improved with medication discontinuation. After appropriate fluid and electrolyte replacement, along with drug discontinuation, rhabdomyolysis resolved. His laboratory tests remained stable, and he was asymptomatic.

There are currently no guideline recommendations of electrolyte monitoring when starting budesonide. This case should prompt physicians to consider checking potassium and creatine phosphokinase on patients who recently started budesonide and complain of muscle aches or excessive fatigue.

## DISCLOSURES

Author contributions: All authors wrote the manuscript. D. Thigpin is the article guarantor.

Financial disclosure: None to report.

Informed consent was obtained for this case report.
